# PEAK RESIDENT SATISFACTION OUTCOMES AND RESEARCH

**DOI:** 10.1093/geroni/igz038.2051

**Published:** 2019-11-08

**Authors:** Judy Poey, Laci Cornelison

**Affiliations:** 1 United Way of Central Maryland, Baltimore, Maryland, United States; 2 Kansas State University, Manhattan, Kansas, United States

## Abstract

Outcomes related to person-centered care in nursing homes have been difficult to ascertain. Much of the extant literature has suffered from differing definitions of what it means to be person-centered, variation in the levels of implementation of person-centered care that an organization has achieved, and small sample sizes. The PEAK program provides a unique opportunity to control for these variables across a large sample of nursing homes throughout the state of Kansas. This presentation will discuss the methodological advantages of evaluating the PEAK program and the findings from an evaluation of resident satisfaction in nursing homes at varying levels of implementation of person-centeredness.

